# L'ostéome ostéoïde: à propos d'un cas

**DOI:** 10.11604/pamj.2016.24.132.9703

**Published:** 2016-06-10

**Authors:** Soukaina Wakrim, Abdellatif Siwane, Ousmane Traore, Samira Lezar, Fatiha Essodegui

**Affiliations:** 1 Service de Radiologie Central, CHU Ibn Rochd, Casablanca, Maroc

**Keywords:** Ostéome ostéoïde, TDM, tumeur osseux, Osteoid osteoma, CT scan, bone tumor

## Abstract

L'ostéome ostéoïde est une tumeur osseuse primitive bénigne. Cette tumeur est relativement fréquente, représente 12% de l'ensemble des tumeurs osseuses bénignes et environ 2 à 3% de l'ensemble des tumeurs osseuses. Nous rapportons un nouveau cas d'ostéome ostéoïde confirmé histologiquement. Il s'agissait d'une patiente âgée de 30 ans adressé pour des douleurs chroniques de la cheville. Les radiographies de la cheville face et profil mettent en évidence une plage hétérogène en regard du col du talus sans anomalies des parties molles. La TDM de la cheville a montré la présence d'une lésion nodulaire hypodense au niveau du col du talus avec une réaction ostéosclérotique peu importante.

## Abstract (en)

Osteoid osteoma is a benign primary bone tumor. This tumor is relatively frequent, accounting for 12% of all benign bone tumors and about 2-3% of all bone tumors. We report a new case of osteoid osteoma confirmed histologically. A 30-year old patient was referred for evaluation of chronic ankle pain. Front and lateral radiographs of the ankle showed a heterogeneous lesion located in the neck of the talus without soft tissue abnormalities. The CT scan of the ankle showed a nodular, hypodense lesion located in the neck of the talus with a limited osteosclerotic reaction.

## Introduction

L'ostéome ostéoïde est une tumeur osseuse primitive bénigne. Cette tumeur est relativement fréquente, représente 12% de l'ensemble des tumeurs osseuses bénignes et environ 2 à 3% de l'ensemble des tumeurs osseuses. Elle touche particulièrement l'adulte jeune avec prédominance masculine. La répartition sur le squelette fait apparaître une prédominance pour les os longs, notamment fémur et tibia (75% des localisations). Le développement de ce type de tumeur sur le col du talus est rare, environ 2% des cas mais caractéristique au niveau du pied. Le traitement curatif est essentiellement chirurgical, l'exérèse complète de la tumeur permet la guérison avec un risque de récidive exceptionnel. Nous rapportons une localisation rare d'ostéome ostéoïde au niveau du talus dont l'aspect scannographique est inhabituel afin d'illustrer l'importance du retentissement fonctionnel d'une telle localisation et les difficultés diagnostiques qui en résultent.

## Patient et observation

**Figure 1:**
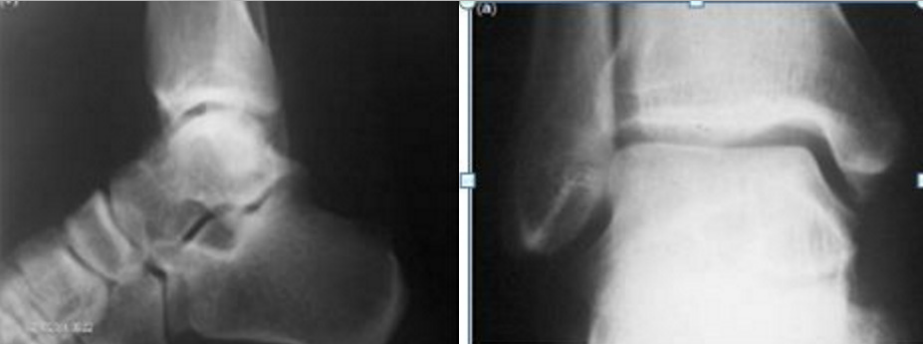
Radiographie de la cheville face et profil évidence une plage hétérogène en regard du col du talus

**Figure 2:**
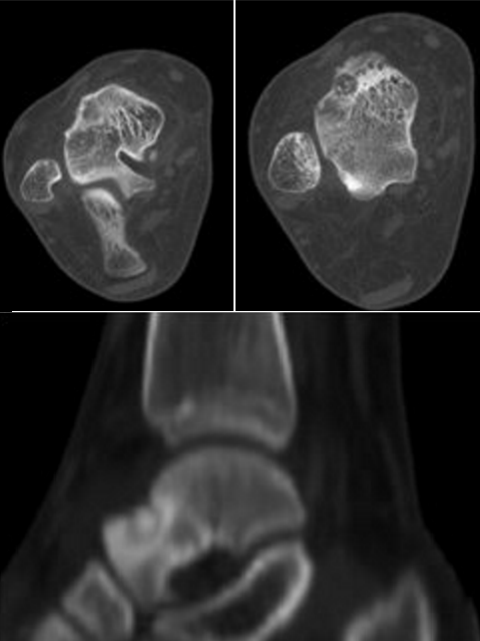
TDM de la cheville: présence d'une lésion nodulaire hypodense au niveau du col du talus avec une réaction ostéo-sclérotique peu importante

**Figure 3:**
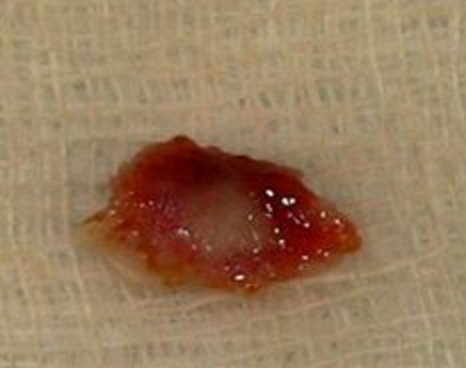
Pièce opératoire

Une jeune femme, âgé de 30 ans, nous a été adressé pour des douleurs chroniques de la cheville remontant à six mois. La douleur persistait malgré les différents traitements médicamenteux et physiques suivis. L'examen clinique de notre patiente a noté une cheville douloureuse, sans point douloureux hyperalgique particulier, et sans anomalie tendineuse ni de la mobilité articulaire. Le bilan biologique n'avait pas montré d'anomalies, en particulier une VS à 10 mm/h et une sérologie rhumatoïde négative. Les radiographies de la cheville face et profil mettent en évidence une plage hétérogène en regard du col du talus sans anomalies des parties molles (Figure 1). La TDM de la cheville a montré la présence d'une lésion nodulaire hypodense au niveau du col du talus avec une réaction ostéosclérotique peu importante (Figure 2). Cette présentation scannographique est inhabituelle pour le talus. Le diagnostic a été soulevé et confirmé histologiquement (Figure 3). La particularité de cette présentation résulte donc de cette forme inhabituelle au niveau du talus.

## Discussion

L'ostéome ostéoïde se caractérise par une structure spécifique, le nidus, entouré d'une ostéocondensation réactionnelle et représente 12% de l'ensemble des tumeurs osseuses bénigne [1]. La localisation au niveau du talus est rare, pouvant ainsi conduire à un retard diagnostique. Snow et al. [2] ont montré dans leur série de cinq patients présentant un ostéome ostéoïde du talus, une moyenne de 2,5 ans pour poser le diagnostic. Le diagnostic est difficile à poser; il faut savoir y penser devant une douleur chronique de la cheville et dont l'imagerie n'est pas toujours spécifique. Néanmoins, le fait que la douleur soit soulagée par la prise de salicylés est un argument majeur en faveur du diagnostic. Une origine tumorale doit toujours être évoquée devant des douleurs chroniques de la cheville a fortiori s'il existe un antécédent de traumatisme, car c'est fréquemment dans ce cas que l'on explique, à tort, les douleurs par des lésions post-traumatiques. L'imagerie classique (radiographie standard et tomodensitométrie [TDM]) évoquait un processus traumatique ou une réaction à un traumatisme plus qu’à une pathologie tumorale. La scintigraphie osseuse ne retenait pas, à tort, par son caractère non fixant de façon élective, le diagnostic d'ostéome ostéoïde [3]. Le diagnostic d'ostéome ostéoïde à scintigraphie négative est exceptionnel. Les hypothèses principalement retenues sont un défaut technique ou une faible activité ostéoblastique de la tumeur [4]. En effet, le nidus se présente comme un petit foyer d'hyperfixation bien circonscrit. Il capte intensément les traceurs radioactifs isotopiques et de manière plus marquée que l'ostéogenèse périphérique.

S'il existe une faible activité ostéoblastique du nidus, on ne trouvera plus l'image en double halo équivalente au nidus radiologique. L'IRM s'est avérée être l'examen le plus sensible. La négativité de la TDM est probablement due à un défaut technique du fait de coupes pas assez jointives. La TDM est, d'après les données de la littérature, l'examen le plus performant [5]. L'IRM est l'examen le plus sensible pour le diagnostic d'ostéome ostéoïde [3, 6] car, elle montre les remaniements de l'os spongieux intramédullaire (oedème intraosseux) et des parties molles périlésionnelles en rapport avec la synovite. Ces lésions apparaissent hyperintenses dans les séquences pondérées en T2 et en saturation de graisse [2, 3]. Assoun et al. [7] ont montré une corrélation significative entre la présence ou l'absence des remaniements de l'os spongieux et des parties molles périlésionnelles visibles sur l'IRM avec la prise d'un traitement anti-inflammatoire. La réaction oedémateuse qui intéresse l'os spongieux ou les parties molles extra osseuses, souvent plus étendue que l'ostéosclérose réactionnelle. Le nidus se rehausse après injection intraveineuse de gadolinium démontrant l'hypervascularisation de la lésion [7, 8, 9] tandis que la sclérose périlésionnelle et la partie centrale calcifiée du nidus apparaissent hypo-intenses dans toutes les séquences [6, 7]. Le non-rehaussement de la zone centrale du nidus avec l'absence de formation abcédée visible permet de la différencier d'une pathologie infectieuse [10]. Devant une suspicion d'ostéome ostéoïde, le bilan d'imagerie doit donc associer une IRM et une TDM en coupes fines centrées sur la lésion. Chacun de ces deux examens amenant des informations utiles au diagnostic positif final [11]. Il précisera aussi la localisation, la taille ainsi que les rapports de la lésion avec les autres structures, notamment les surfaces articulaires. Il permet de planifier le traitement chirurgical qui consiste en l'excision de la totalité du nidus pour prévenir les récidives [7, 8].

## Conclusion

Les ostéomes ostéoïdes du talus sont rares. La symptomatologie est trompeuse. En cas de doute diagnostique et devant des radiographies normales, l'examen le plus spécifique est la tomodensitométrie en coupes fines, dont la sensibilité peut être améliorée par l'association à une IRM. L'imagerie permet ainsi d'affirmer le diagnostic avant la cure chirurgicale de la lésion qui doit être complète.
